# Supplementary material for: Comparative genomics provides new insights into the diversity, physiology, and sexuality of the only industrially exploited tremellomycete: Phaffia rhodozyma
Source: BMC Genomics. 2016 Nov 9;17:901. doi: 10.1186/s12864-016-3244-7 (PMC5103461; doi:10.1186/s12864-016-3244-7)
Supplement: Additional file 6: — List of orphan genes with links to PFAM (related to Additional file 1: Table S1). (ZIP 1428 kb) [file 12864_2016_3244_MOESM6_ESM.zip › PFAM_UNIQUE/G05174_P.html]

#                                                                            --- full sequence --- -------------- this domain -------------   hmm coord   ali coord   env coord
  
# target name        accession   tlen query name           accession   qlen   E-value  score  bias   #  of  c-Evalue  i-Evalue  score  bias  from    to  from    to  from    to  acc description of target
  
#------------------- ---------- ----- -------------------- ---------- ----- --------- ------ ----- --- --- --------- --------- ------ ----- ----- ----- ----- ----- ----- ----- ---- ---------------------
  
G05174\_P             -            240 BLVR                 PF06375.6    154       4.5    5.7   9.2   1   1     0.078        31    3.0   9.2    18   102   150   229   111   238 0.58 - Bovine leukaemia virus receptor (BLVR)  
G05174\_P             -            240 U3\_snoRNA\_assoc      PF08297.6     88      0.27   10.2   4.3   1   1   0.00038      0.46    9.4   4.3     9    52   187   232   180   239 0.70 - U3 snoRNA associated
